# Cerebroprotective effects of *Moringa oleifera* derivatives extracts against MCAO ischemic stroke: A systematic review and meta-analysis

**DOI:** 10.1016/j.heliyon.2023.e16622

**Published:** 2023-05-29

**Authors:** Andea Cuschieri, Emma Camilleri, Renald Blundell

**Affiliations:** University of Malta, Imsida, MSD, 2080, Malta

**Keywords:** Moringa oleifera, Moringa, Phytochemical, Ischemic stroke, MCAO, Meta-analysis, In-vivo, Stroke, Cerebroprotection

## Abstract

*Moringa oleifera* (MO), has been studied extensively, and has numerous medicinal and socioeconomic benefits. Emerging research has investigated the efficacy of MO extract and/or its phytochemical derivatives against ischemic stroke *in-vivo*. To date, no studies comprehensively reviewing the effects of MO extract and/or its phytochemical derivatives against ischemic stroke have been published. A systematic review and meta-analysis was conducted to assess the effects of MO extract and/or its phytochemical derivatives against focal ischemic stroke, modeled *in-vivo*. Compared with control groups, significant reduction in infarct volume and malondialdehyde levels, and signficant increase in antioxidant enzymes superoxide dismutase, glutathione peroxidase and catalase. The primary mechanism of action of MO extract and its phytochemical derivatives which confers neuroprotection is reduction in oxidative stress by increasing antioxidant enzymes. On the whole, the present systematic review critically assessed evidence which demonstrated that MO extract may confer protective effect on experimental ischemic stroke. Although effect size may have been overestimated due to the limited number of included studies, small sample sizes and possible publication bias, results generated in this meta-analysis dmeonstrate that MO extract may be a promising neuroprotective agent against human ischemic stroke.

## 1 Introduction

**Fig. 1:**
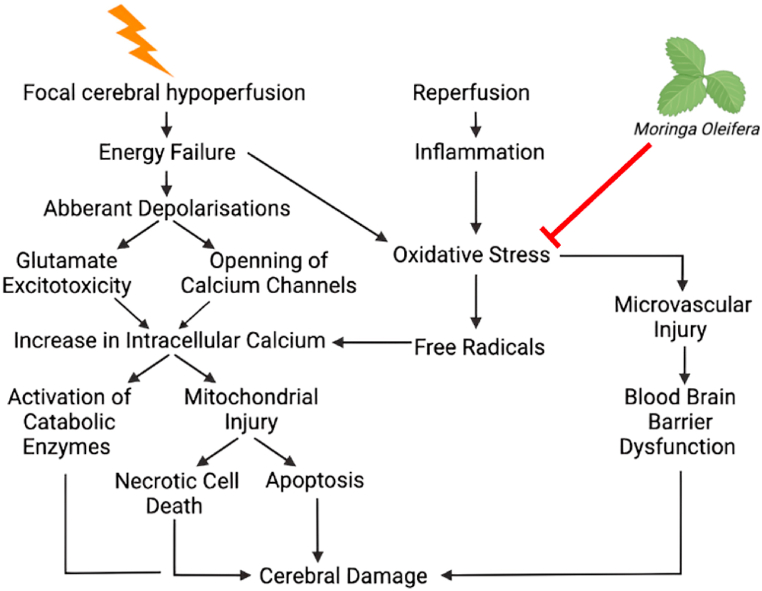
Hypothesised effect of Moringa oleifera on the ischemic cascade [[15], [16], [17], [18]]. Created witg BioRender.com.

Ischemic stroke is the most prevalent form of stroke, accoutning for 62.4% of all incident stroke events representing approcimately 11 million people annually and 4% of the total health care expendirue in western countries [[1], [2], [3]]. The pathological mechanisms governing ischemic stroke are complex, involving numerous cellular mechanisms [4]. The clinical use of current treatments for ischemic stroke are limited due to patient contraindications and strict timing criteria [5]. Moreover, therapeutic approaches which confer neuroprotection against ischemic stroke are scarce [6]. Hence, given that ischemic stroke is major health care burden and limited therapeutic approaches, exploration of putative pharmacological treatment modalities is warranted. Natural extracts and phytochemicals have recently received extensive attention as an indispensable resource for drug discovery for their long history of clinical application [7]. Indeed, numerous extracts and compounds have been identified to have promising efficacy against ischemic stroke [8]. Considering the complexity of stroke pathophysiology, it is necessary to investigate all possible extracts/compounds which may confer neuroprotection against ischemic stroke. *Moringa oleifera* (MO), is an extensively studied plant due its ability to grow under a myriad of environmental conditions [[9], [10], [11]], as well as its various applications in medicinal and socioeconomic sectors [12]. MO is also a rich source of a wide range of phytochemicals, which, when purified, each have their own unique medicinal properties [13]. Whilst its phytochemistry is has been largely investigating for managing diseases like diabetes mellitus and hypertension [14], recent studies have suggested that MO's derivatives may act as promising neuroprotective agents against ischemic stroke but reducing the oxidative stress [[15], [16], [17], [18]] (Fig. 1).

Systematic reviews and meta-analysis are a form of secondary research which compile all primary research which meet prespecified stringent qualifying criteria, to answer a specific research question whilst minimizing bias. They can provide necessary evidence to help guide the choice of optimal drug administration requirements in clinical trials [19]. Reviews have been published documenting the effects of MO on the nervous system [17,[20], [21], [22]], which have highlighted the potential cerebroprotective effects of MO against ischemic stroke. Yet, an in-depth understanding of current understanding on the putative protective effects of MO against ischemic stroke stemming from studies which investigate the effect of MO extracts or the plants’ phytochemical derivatives on focal ischemic stroke models is unknown [17,20]. Here, a systematic review and meta-analysis is conducted to appraise the effectiveness and therapeutic outcomes of MO extracts or phytochemical derivatives against MCAO models of ischemic stroke.

## 2 Methods

This systematic review and meta-analysis was carried according to the Preferred Reporting items of systematic reviews and meta-analyses (PRISMA) guidelines (Supplementary material A) and the Cochrane handbook for systematic reviews of interventions [23,24]. The Equator network was consulted to identify appropriate quality assessment and risk of bias (RoB) tools [25].

### 2.1 Database search strategy

Four major databases were searched: PubMed, Google Scholar, Science Direct and Semantic Scholar data, using combinations of the following keywords: “Moringa oleifera”, “M. oleifera”, “ischemic stroke”, “focal ischemic stroke”, “middle cerebral artery occlusion (MCAO)” and “phytochemical”. Citation searching of relevant articles was conducted. All studies published in English from inception up to February 2023 were collected. There were no restrictions on the country of publication. Grey literature was not explored, since only articles published in reputable peer-reviewed journals were considered. Identified studies were retried from the respective database and stored digitally in dedicated files.

### 2.2 Inclusion criteria

All rodent *in-vivo* experiments assessing the effect of MO oil/extract and/or of any phytochemical significantly present in MO oil/extract [13] on focal cerebral ischemia were included, regardless of animal age and sex. Included studies had to satisfy the following inclusion criteria [1]: MO oil/extract and/or of any phytochemical significantly present in MO oil/extract [13] was administered to a rodent model of focal cerebral ischemia, regardless of route, dosage or treatment regimen [2]; focal ischemia was achieved *in-vivo* using middle cerebral artery occlusion (MCAO) [3]; intervention group(s) only used MO oil/extract or of any phytochemical significantly present in MO oil/extract [13]; and [4], control animals received the same operation and vehicle, without receiving any treatment.

### 2.3 Outcome measures

Primary outcome indicators included infarct volume and Bederson NFS. Secondary outcome indicators were levels of malondialdehyde (MDA), superoxide dismutase (SOD), glutathione peroxidase (GSH-PX) and catalase (CAT) in the cortex, hippocampus and striatum together with any other results reported, which arise as a consequence to MO oil/extract or of any phytochemical significantly present in MO oil/extract [13] administration.

### 2.4 Exclusion criteria

The following exclusion criteria were employed [1]: study was a review, perspective, case report, *in-vitro* experiment, *ex-vivo* study, *in-silico* study, or human study [2]; non-focal cerebral ischemia models such as global cerebral ischemia, hypoxia-ischemia, traumatic models or chronic cerebral ischemia [3]; MO oil/extract or of any phytochemical significantly present in MO oil/extract [13] administered in-combination with other compounds and/or treatments [4], no positive (MCAO + vehicle) control group; and [5], no indication of sample size.

### 2.5 Data extraction

Two independent reviewers independently extracted the data using standardised data-extraction tables. Any disagreements were resolved consensus. The following data was extracted [1]: species and sex of the animals used [2]; weight of the animals used [3]; technique used to generate focal ischemia [4]; anaesthetic protocol [5]; treatment administered [6]; control conditions; and [7] outcome measures.

### 2.6 Quality assessment

Two independent reviewers evaluated the quality of included studies according the combined essential 10 and recommended set of Animal Research: Reporting of In Vivo Experiments (ARRIVE) guidelines 2.0, established by the National Centre for the Replacement, Refinement and Reduction of Animals in Research [26]. Any disagreements were resolved consensus.

### 2.7 Risk of bias evaluation

RoB for each included study was evaluated by employing the SYRCLE RoB tool for animal studies [27]. Two independent reviewers assessed the RoB of the included studies across across domains for sequence generation (Selection bias), baseline characteristics (Selection bias), allocation concealment (Selection bias), random housing (Performance bias), blinding of personnel and outcome assessors (Performance and detection bias), random outcome assessment (Detection bias), incomplete outcome data (Attrition bias), selective outcome reporting (Reporting bias). Each domain was afforded one of three possible judgements: “low risk of bias”, “high risk of bias” or “unclear risk of bias”. Any disagreements were resolved consensus.

### 2.8 Statistical analysis

#### 2.8.1 Assessment of heterogeneity

Heterogeneity amongst included studies was conducted by examining forest plots for overlap of confidence intervals (CI). Statistical heterogeneity through *Chi*^*2*^ testing (*p*-value <0.10) and quantified through *I*^2^ statistic.

#### 2.8.2 Data synthesis

Microsoft excel® (version 16.70) was used to calculate mean difference, Cohen's D and pooled standard error. α-symbol was set at 0.05. JASP (version 0.15) software and meta-analysis add-on were used to generate forest plots and compute CIs. Fixed-effects model was employed without statistical evidence of heterogeneity (*p* ≥ 0.1, *I*^2^ ≤ 50%). Residual maximum likelihood (REML) model was employed in instances of statistical heterogeneity (*p* < 0.1, *I*^2^ > 50%).

### 2.9 Assessment of publication bias

Publication bias was assessed visually using funnel plots, generated using JASP (version 0.15) software and meta-analysis add on, and Kendall's τ and Egger's test computed using the same software.

## 3 Results

### 3.1 Study selection

**Fig. 2:**
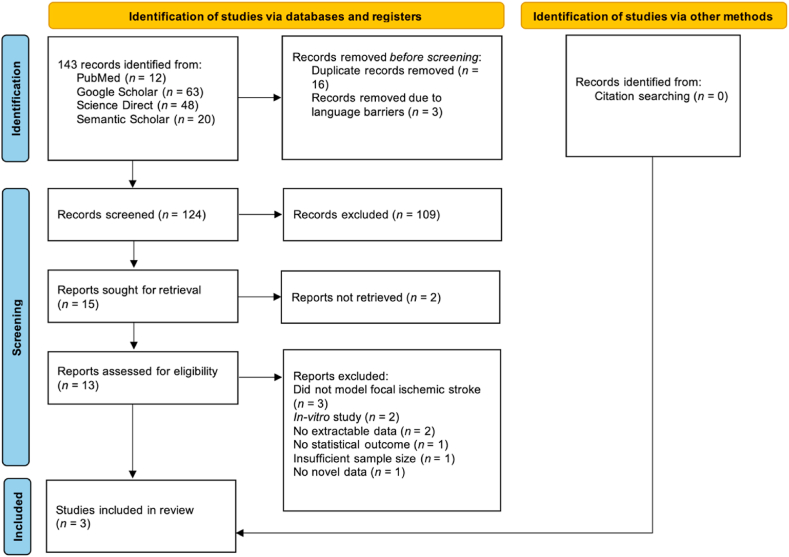
Prisma 2020 flow diagram. Adapted from Page et al. (2021).

143 tentative studies were identified, with 16 potential records being duplicates. 109 studies were excluded following title and abstract screening using the pre-defined inclusion criteria. Thus, 15 reports were sought for retrieval and their full text was assessed in detail for eligibility. Among these, 2 studies could not be retrieved and 10 studies were excluded following full-text review, due to the following reasons [1]: in-vitro study [2]; no extractable data [3], did not model focal ischemic stroke [4]; no statistical outcome [5]; insufficient sample size; and [6], no novel data was reported. Citation-searching did not reveal any additional studies which met out inclusion criteria, hence 3 studies were included in this systematic review and meta-analysis [[28], [29], [30]]. Fig. 2 demonstrates the screening process utilised in this systematic review and meta-analysis.

### 3.2 Characteristics of included studies

Between 2013 and 2023, three studies were published, two of which included Wistar rats [28,29], while utilised on Sprague Dawley (SD) rats and Kumming (KM) mice [30]. Khoshnazar et al. (2019) investigated the effects a phytochemical derived from MO, α-pinene, while Kirisattayakul et al. (2013) and Zeng et al. (2019) investigated the effects of ethanolic MO extract against ischemic stroke. Two studies employed chloral hydrate [29,30], while Kirisattayakul et al. (2013) employed thiopental sodium to achieve anaesthesia. Focal cerebral ischemia was achieved by right MCAO in all included studies. Moreover, all studies investigated dose gradient of the treatment employed, and administered treatment prior to ischemia induction. Zeng et al. (2019) also investigated the effects of post-MCAO treatment administration on the aftermath of ischemic stroke. All include studied reported infarct volume post MCAO and Bederson neurological function scores (NFS). Full details about the characteristics of the included studies are demonstrated in Table 1.

**Table 1:** Characteristics of the included studies.

Study	Species (sex)	Weight (g)	Global ischemia model	Aesthetic protocol	Extract/phytochemical preparation	Treatment	Control	Outcome index
Kirisattayakul et al. (2013)	Wistar rats (male)	300–350	rt. MCAO	Thiopental sodium dosed at 60 mg/kg BW i.p.	50% hydroalcohol extraction, filtered, from dried fresh leaves using maceration technique	(a) 100 mg/kg BW oral MO; (b) 200 mg/kg BW oral Me; (c) 400 mg/kg BW oral MO; over a duration of 14 days + MCAO	Oral 1% carboxymethyl-cellulose + MCAO	(1) Infarct volume (TCC), 24 h after MCAO (5/5) (2) NFS (Bederson), 7, 14, 21 days after MCAO (5/5) (3) Sensory function (hot plate test), 7, 14, 21 days after MCAO (5/5) (4) MDA, (cervical dislocation) 24 h after MCAO (5/5); in cortex hippocampus and striatum (5) SOD, 24 h after MCAO (5/5); in cortex hippocampus and striatum (6) GSH-Px, 24 h after MCAO (5/5); in cortex hippocampus and striatum (7) CAT, 24 h after MCAO (5/5); in cortex hippocampus and striatum
Khoshnazar et al. (2019)	Wistar rats (male)	250–300	rt. MCAO	Chloral hydrate dosed at 300 mg/kg BW i.p.	α-pinene was purchased from Sigma-Aldrich (St. Louis, Mo, USA)	(a) MCAO + 25 mg/kg BW i.p. α-pinene (b) MCAO + 50 mg/kg BW i.p. α-pinene (c) MCAO + 100 mg/kg BW i.p. α-pinene; immediately after reperfusion	Mixture of normal saline and tween 80	(1) Infarct volume (TCC), 24 h after MCAO (5/5) (2) NFS (Bederson), 24 h after MCAO ((5/5) (3) Oedema 24 h after MCAO (5/5) (4) MDA, (cervical dislocation) 24 h after MCAO (5/5); in cortex hippocampus and striatum (5) SOD, 24 h after MCAO (5/5); in cortex hippocampus and striatum (6) GSH-Px, 24 h after MCAO (5/5); in cortex hippocampus and striatum (7) CAT, 24 h after MCAO (5/5); in cortex hippocampus and striatum (8) NO, 24 h after MCAO (5/5); in cortex hippocampus and striatum (9) Il-6, 24 h after MCAO (5/5); in cortex hippocampus and striatum
Zeng et al. (2019)	SD rats (male) and KM mice (n.a.)	250–500 g and 25–30	rt. MCAO	Chloral hydrate dosed at 400 mg/kg BW i.p.	70% hydroalcohol extraction, filtered, from dried seeds	(a) 125 mg/kg BW oral MO; (b) 250 mg/g BW oral MO; (c) 500 mg/kg BW oral MO; over a duration of 3 days + MCAO (d) MCAO + 500 mg/kg BW oral MO, 0 h after MCAO; (e) MCAO + 500 mg/kg BW oral MO, 2 h after MCAO; (f) MCAO + 500 mg/kg BW oral MO, 4 h after MCAO (g) MCAO + chronic oral MO treatment (500 mg/kg BW oral MO from day 1 up to 21 after MCAO)	Saline (10% v/v)	(1) Infarct volume (TCC), 24 h after MCAO (12/12) (2) NFS (Bederson), 24 h after MCAO (12/12) (3) Oedema (TCC), 24 h after MCAO (12/12) (4) Neuroprotection and survival rate, 1–21 days after MCAO (12/12) (5) Spatial learning and memory, 21 days after MCAO (6) Neurogenesis (administration of BrdU (50 mg/kg) days 1–7 post MCAO, BrdU and NeuN markers, hippocampal and cortical GAP-43, SYP), 24 h after MCAO (6/12) (7) Hippocampal BDNF, NT3 and NGF mRNA expression, 24 h after MCAO (4/12 kM mice) (8) Hippocampal Ach, AChE and ChAT protein expression 24 h after MCAO (4–5/12 SD rats)

### 3.3 Study quality evaluation

**Fig. 3:**
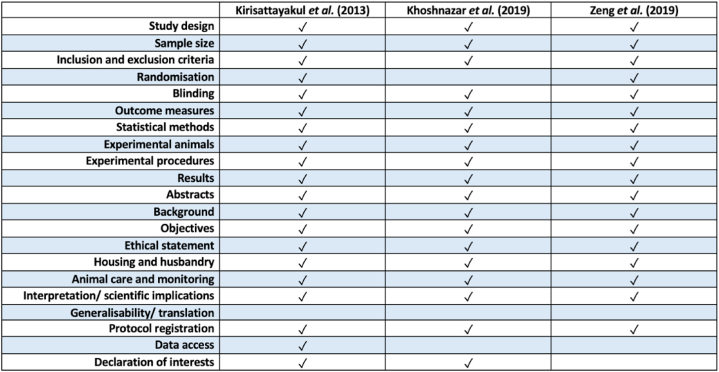
Study quality evaluation following ARRIVE criteria.

A 21 point scoring method was used to evaluate the quality of included studies (Fig. 3). The study quality ranged between 16 and 18 points points, with an average of 16.67 points. All included studies were peer-reviewed publications. All studies adequately reported study design, sample size, inclusion and exclusion criteria, statistical methods, experimental procedures and results. Moreover, all studies had well defined outcome measures, but none reported taking measures to randomise their experiments. All studies report compliances with animal welfare regulations. Data accessibility and explanations of generalisability of generated results were lacking across all included studies.

### 3.4 Risk of bias assessment

**Fig. 4:**
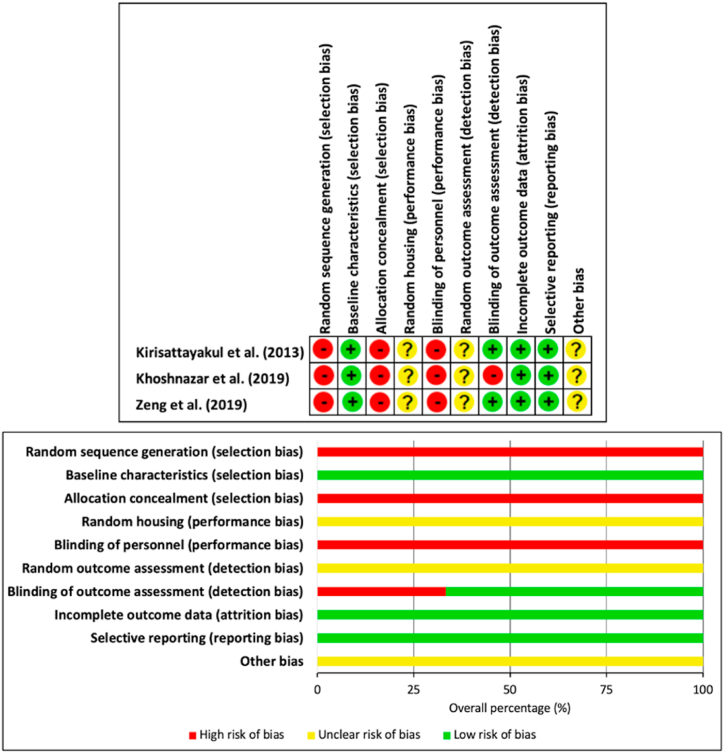
Reporting risk of bias using SYCLE criteria.

Measures taken to adequately address and reduce RoB was similar across all included studies (Fig. 4). No studies reported using techniques of random sequence generation, allocation concealment or blinding of personnel to interventions. Unclear random housing and random outcome assessment bias was not across all included studies. RoB was most commonly minimised by reporting baseline characteristics, blinding of personnel during outcome assessment, having complete data and unselective reporting.

### 3.5 Assessment of effectiveness

#### 3.5.1 Infarct volume

**Fig. 5:**
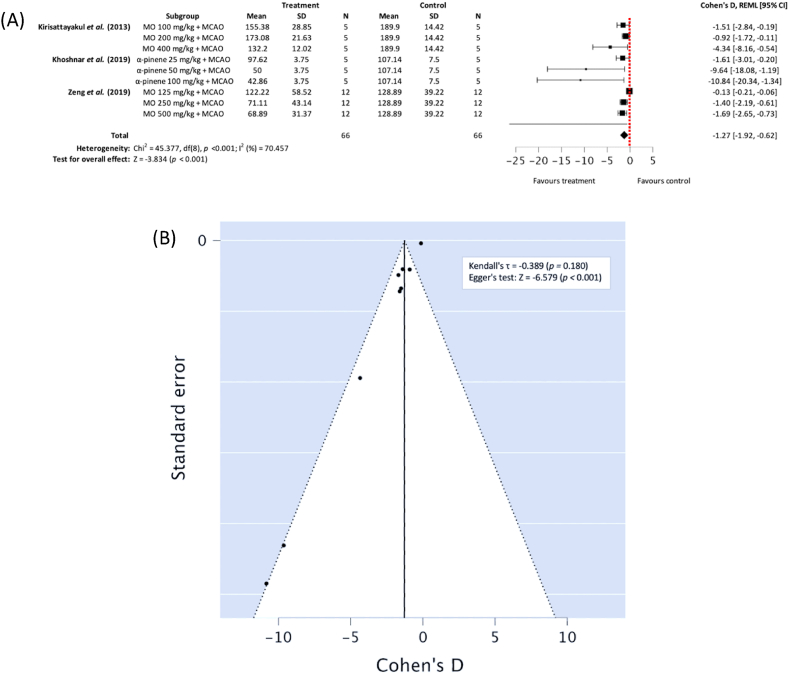
(A) Forest plot demonstrating the effects of different concentration *Moringa oleifera* ethanolic extract and α-pinene for reducing infract volume in comparison with control groups, (B) funnel plot assessing publication bias (MO – Moringa oleifera, MCAO – middle cerebral artery occlusion, REML - residual maximum likelihood, CI – confidence intervals).

Subgroup meta-analysis of the three included studies [[28], [29], [30]] demonstrated that ethanolic MO extract and α-pinene have a promising effect in reducing ischemic infarct volume in comparison to vehicle + MCAO control group, with higher concentrations of α-pinene having the greatest effect at reducing infarct volume (*n*_*T*_*/n*_*C*_ = 66/66), Cohen's D: −1.27, 95% CI [−1.92, −0.62], *p <* 0.001; heterogeneity: Chi^2^ = 45.377, df = 8 (*p* < 0.001); *I*^*2*^ = 70.457% (Fig. 5A). Egger testing demonstrated publication bias may have been present (*p* < 0.001) (Fig. 5B).

#### 3.5.2 Neurological function scores

**Fig. 6:**
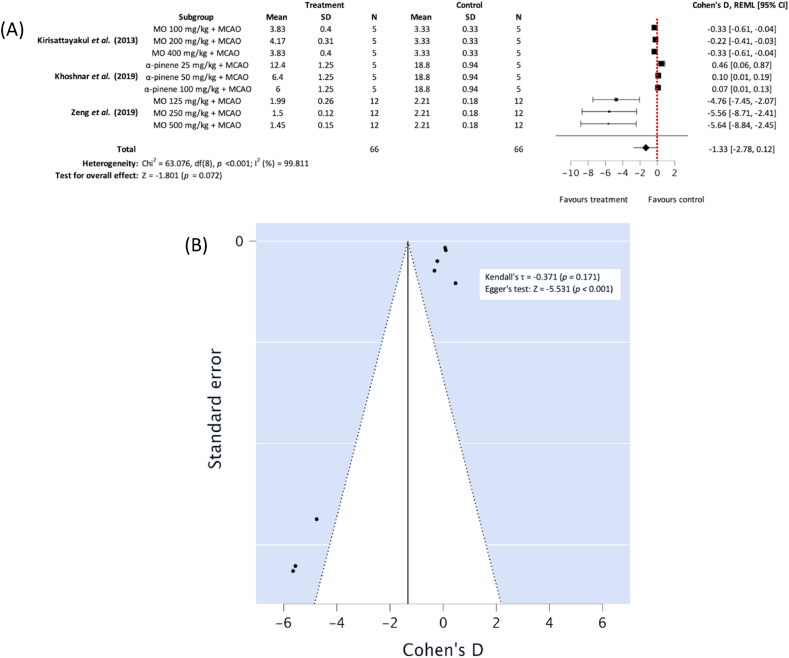
(A) Forest plot demonstrating the effects of different concentration *Moringa oleifera* ethanolic extract and α-pinene on neurological function scores in comparison with control groups, (B) funnel plot assessing publication bias (MO – Moringa oleifera, MCAO – middle cerebral artery occlusion, REML - residual maximum likelihood, CI – confidence intervals).

The three included studies [[28], [29], [30]] reported NFS according to the Bederson criteria. Subgroup meta-analysis showed MO ethanolic favoured treatment conditions in increasing post-MCAO ischemia NFS, however this was not statistically significant, possibly due to α-pinene administration favouring control (*n*_*T*_*/n*_*C*_ = 66/66), Cohen's D: −1.33, 95% CI [−2.98, 0.12], *p* = 0.072; heterogeneity: Chi^2^ = 63.076, df = 8 (*p* < 0.001); *I*^*2*^ = 99.811% (Fig. 6A). Egger testing demonstrated publication bias may have been present (*p* < 0.001) (Fig. 6B).

#### 3.5.3 Cerebral oedema

**Fig. 7:**
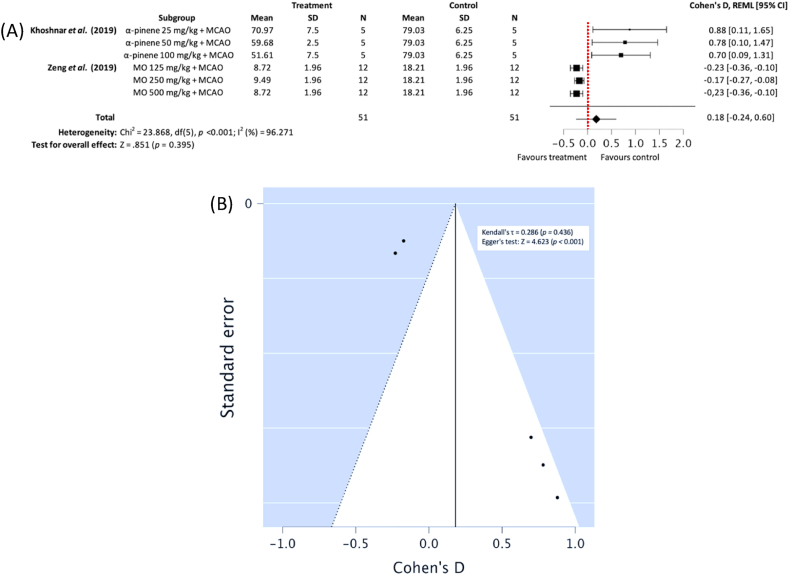
(A) Forest plot demonstrating the effects of different concentration *Moringa oleifera* ethanolic extract and α-pinene on cerebral oedema in comparison with control groups, (B) funnel plot assessing publication bias (MO – Moringa oleifera, MCAO – middle cerebral artery occlusion, REML - residual maximum likelihood, CI – confidence intervals).

Subgroup meta-analysis of two studies [29,30] demonstrated statistically insignificant effect against post-MCAO ischemic cerebral oedema. However, it should be noted that MO ethanolic extract administration favoured treatment conditions (reducing oedema) while α-pinene favoured control conditions (no effect on oedema) (*n*_*T*_*/n*_*C*_ = 51/51), Cohen's D: 0.18, 95% CI [−0.36, −0.10], *p* = 0.395; heterogeneity: Chi^2^ = 23.868, df = 5 (*p* < 0.001); *I*^*2*^ = 96.271% (Fig. 7A). Egger testing demonstrated publication bias may have been present (*p* < 0.001) (Fig. 7B).

#### 3.5.4 Malondialdehyde and antioxidant enzyme levels

**Fig. 8:**
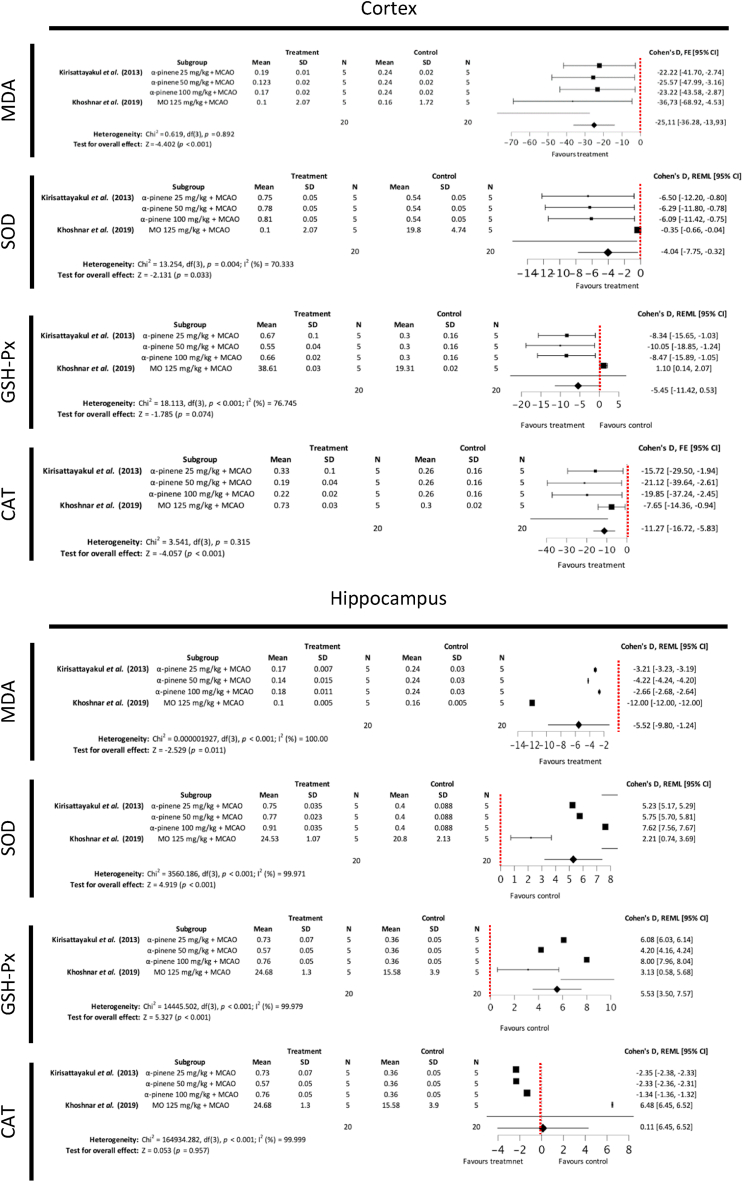

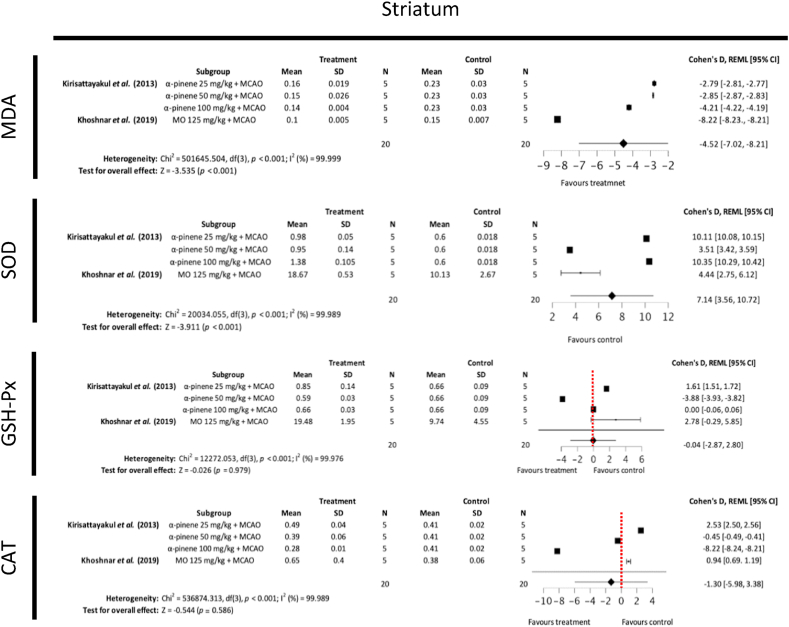
Forest plots demonstrating the effects of different concentration *Moringa oleifera* ethanolic extract and α-pinene on cortical, hippocampal and striatal malondialdehyde, superoxide dismutase, glutathione peroxidase and catalase levels in comparison to control groups (MO – Moringa oleifera, MCAO – middle cerebral artery occlusion, FE – fixed effects, REML - residual maximum likelihood, CI – confidence intervals).

**Fig. 9:**
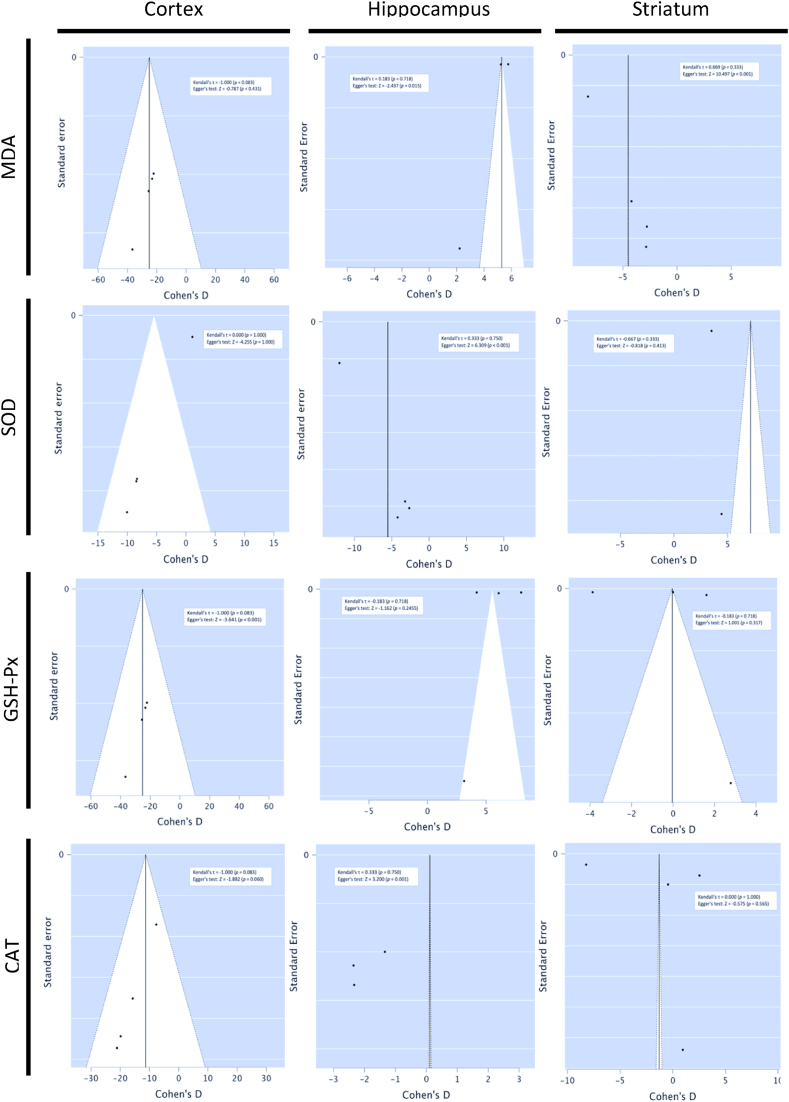
Funnel plots assessing publication bias for subgroup meta-analysis of MDA, SOD, GSH-Px and CAT in the cortex, hippocampus and striatum.

MDA, SOD, GSH-Px and CAT levels in the rodent cerebral cortex, hippocampus and striatum post-MCAO ischemia were reported as secondary outcome measures in two included studies [28,29]. MDA is an end product of polyunsaturated fatty acid peroxidation and widely recognised as a marker of oxidative stress [31]. SOD, GSH-Px and CAT are among the most important antioxidant enzymes, which confer protection against ischemia [32]. Subgroup meta-analysis showed that treatment with MO ethanolic extract or its phytochemical derivative α-pinene demonstrated statistically significant reduction in MDA levels in the cortex, hippocampus and striatum, increase of SOD in the cortex, hippocampus and striatum, increase in CAT in the cortex and increase of GSH-Px in the hippocampus (Fig. 8). Publication bias was noted for meta-analyses assessing levels of SOD in the cortex, MDA and CAT in the hippocampus, and MDA in the striatum (Fig. 9).

## 4 Discussion

### 4.1 Summary of evidence

**Fig. 10:**
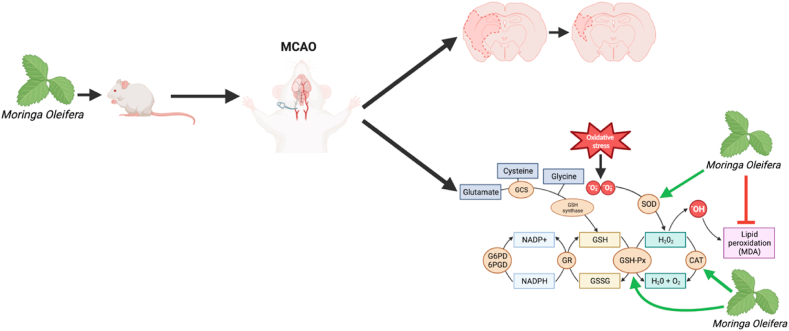
Proposed mode of action of *Moringa oleifera* following focal cerebral ischemia generated by middle cerebral artery occlusion (MCAO – middle cerebral artery occlusion, SOD – superoxide dismutase, MDA – malondialdehyde, GSH-Px – glutathione peroxidase, CAT – catalase, GCS – glutamyl cysteine synthetase, GSSH – oxidised glutathione, GR – glutathione reductase, GSH – glutathione, G6PD – glucose-6-phosphate dehydrogenase, 6PGH – 6-phosphogluconate dehydrogenase, NADPH – nicotinamide adenine dinucleotide phosphate-oxidase, OH – hydroxyl radical, H_2_O_2_ – hydrogen peroxide). Created with BioRender.com.

To date, a total of three studies met our pre-defined inclusion criteria, which assessed the effect of ethanolic MO extract [28,30], and the phyothemical derivative, α-pinene [29], against focal ischemic stroke *in-vivo,* generated through MCAO. The main findings of this meta-analysis demonstrated that MO ethanolic extract and α-pinene have promising potential as neuroprotective agents against ischemic stroke, since they are able to reduce infarct volume and MDA levels while increasing antioxidant enzymes in comparison to controls (Fig. 10). It is worth noting that in a CoCl2 ischemic model, Mohamed et al. (2019) noted an increase in SOD and CAT levels, coupled with decreased MDA levels in MO extract treated mice further demonstrating MO extract's cerebroprotective potential. Such findings consolidate published literature which suggest that phytochemicals increase anti-oxidant enzymes, decreasing ischemic stroke induced oxidative stress conferring neuroprotection [8]. Yet, clinical studies investigating the cerebroprotective effects of natural extracts and phytochemicals against ischemic stroke are limited [[33], [34], [35]], and to date no clinical studies assessing the cereborpotective effects of MO extracts have been conducted. Moreover, rodent MCAO ischemic stroke pathophysiology may be different to that of human ischemic stroke [36], limiting generalisability of findings to the clinical settings. Yet, pre-clinical findings presented in this review give essential evidence which may warrant clinical-trails to investigate the effects of MO extracts and phytochemical derivatives on human ischemic stroke.

### 4.2 Strengths and limitations

This study is the first meta-analysis to systematically scope the effects of MO extract and its phytochemical derivatives against focal cerebral ischemia. In this meta-analysis, four major databases were used with numerous search terms to ensure the extensive retrieval of peer-reviewed published articles. Moreover, stringent inclusion and exclusion criteria were implemented, alongside the use of ARRIVE quality assessment criteria and SYRCLE RoB tools.

This research identified key concepts and sources of evidence on the neuroprotective effects of MO extracts and its phytochemical derivatives against focal ischemic stroke which has practical value for evidence-based transformation of animal data to clinical research.

This systematic review also had some limitations. The limited number of included studies may limit generalisability of results. However, the field of research scoped by this study is emergent, giving context to the limited studies available. Since included studies were in English, this may have led to selection bias. Moreover, no studies reported the negative effects of MO extracts and its phytochemical derivatives against focal ischemic stroke, which may be a key contribution to publication bias. In addition, methodological quality of included studies lacked sufficient randomisation, average blinding and no sample size calculations. Therefore, some conclusions in the present study should be inferred critically.

### 4.3 Implications of findings

This meta-analysis identified MO extract and its phytochemical derivatives as promising agents which may confer neuroprotection against ischemic stroke. Moreover, as previously discussed [17], this systematic review has further consolidated the current paradigm that increases in antioxidant enzymes is a putative primary mechanism of which confers cerebroprotection against ischemic stroke. High quality methodology is the corner stone of translating animal research into clinical trials for human diseases [37]. The methodological quality of included studies was average due to lack of sample size estimation, adequate randomisation, variable blinding across studies. Insufficient sample size may diminish the effect of interventions, however large sample sizes may lead to animal waste and result in ethical concerns. In addition, animals with relevant comorbidities were not used to investigate the effects of MO extract and its phytochemical derivatives which do not truly reflect human pathology under clinical conditions. Methodological quality may be improved by utilizing the ARRIVE criteria [26].

## 5 Conclusion

MO ethanolic extracts and its phytochemical derivative, α-pinene, may reduce cerebral infarct volume and MDA levels, while conferring further cereborpotective effects by increasing the levels of antioxidant enzymes in SOD, GSH-Px and CAT, which have the potential to reduce oxidative stress generated by ischemic stroke. Naturally derived extracts and phytochemicals may be promising agents in the discovery of putative cerebroprotective agents against ischemic stroke since they may upregulate antioxidant enzymes, which are key-modulators of ischemic injury. Further studies are necessary to consolidate findings concluded in this study and identify other currently unknown molecular mechanisms governing the cerebroprotective effects MO and other naturally derived extracts.

## Author contribution statement

All authors listed have significantly contributed to the development and the writing of this article.

## Data availability statement

Data included in article/supplementary material/referenced in article.

## Declaration of competing interest

The authors declare that they have no known competing financial interests or personal relationships that could have appeared to influence the work reported in this paper.
